# Sex differences in the effect of childhood adversity and coping strategies on psychosis expression: A TwinssCan study

**DOI:** 10.1192/j.eurpsy.2026.10173

**Published:** 2026-02-20

**Authors:** Melike Karaçam Doğan, Thanavadee Prachason, Laura Fusar-Poli, Claudia Menne-Lothmann, Jeroen Decoster, Ruud van Winkel, Dina Collip, Philippe Delespaul, Marc De Hert, Catherine Derom, Evert Thiery, Nele Jacobs, Marieke Wichers, Bart P. F. Rutten, Jim van Os, Lotta Katrin Pries, Sinan Guloksuz

**Affiliations:** 1 Department of Psychiatry, Ankara Bilkent City Hospital, Ankara, Türkiye; 2 Department of Psychiatry and Neuropsychology, Maastricht University School for Mental Health and Neuroscience, Maastricht, Netherlands; 3 Department of Psychiatry, Mahidol University Ramathibodi Hospital, Bangkok, Thailand; 4 Department of Brain and Behavioral Sciences, University of Pavia, Pavia, Italy; 5 Istituti Clinici Scientifici Maugeri IRCCS, Pavia, Italy; 6 KU Leuven Psychiatric University Hospital, Leuven, Belgium; 7 Department of Obstetrics and Gynecology, Ghent University Hospital, Ghent, Belgium; 8 Department of Neurology, Ghent University Hospital, Ghent, Belgium; 9 Faculty of Psychology, Open University of The Netherlands, Netherlands; 10 Department of Psychiatry, Interdisciplinary Center Psychopathology and Emotion Regulation, University Medical Centre Groningen, Groningen, Netherlands; 11 Division Neuroscience, UMC Utrecht, Utrecht, Netherlands; 12 Department of Psychosis Studies, Institute of Psychiatry, King’s Health Partners, King’s College London, London, UK; 13 University of Cologne, Faculty of Medicine and University Hospital Cologne, Department of Psychiatry and Psychotherapy, Cologne, Germany; 14 Department of Psychiatry, Yale School of Medicine, New Haven, CT, USA; 15 Department of Psychiatry, University of British Columbia, Vancouver, BC, Canada; 16 Institute of Mental Health, University of British Columbia, Vancouver, BC, Canada; 17 Northern Medical Program, Division of Medical Sciences, University of Northern British Columbia, Prince George, BC, Canada

**Keywords:** childhood adversity, coping strategies, general population, psychosis expression, sex differences

## Abstract

**Background:**

Sex differences in psychosis pathoetiology are insufficiently understood. This study explores how childhood adversity (CA) and coping mechanisms relate to psychosis expression (PE) across males and females in the general population.

**Methods:**

Data from the TwinssCan project (males: *n* = 312; females: *n* = 478) were used. The Childhood Trauma Questionnaire assessed CA domains. The Utrecht Coping List assessed coping strategies. Psychosis expression was assessed using the Community Assessment of Psychic Experiences (CAPE). Mixed linear regression analyses examined sex-stratified associations of CAPE scores with CA, coping strategies, and their interactions.

**Results:**

Emotional abuse (EA) was associated with increased total CAPE scores (T-CAPE), explaining the greatest variance among CA across sexes. Sex-specific effects showed that sexual abuse (SA) and physical abuse (PA) were linked to higher T-CAPE in females, whereas physical neglect (PN) was linked to higher T-CAPE in males. Passive-reacting was associated with increased T-CAPE, explaining the greatest variance among coping styles across both sexes. Sex-specific effects showed that, in females, seeking social support was linked to decreased T-CAPE, while emotional expression increased it. The only sex-shared interaction effect was between reassuring thoughts and emotional neglect (EN), associated with decreased T-CAPE. In females, social support (× PA/PN/EA), reassuring thoughts (× PA/PN), and palliative-reacting (× PN/PA) were associated with decreased T-CAPE, while passive-reacting (× EN) increased it. In males, avoidance (× SA/PA) and passive-reacting (× PN) were associated with increased T-CAPE.

**Conclusions:**

Sex differences in the associations of PE with CA and coping underscore the necessity for sex-specific interventions that promote adaptive coping strategies.

## Introduction

Psychosis expression (PE) in the general population can precede the development of severe psychotic and non-psychotic disorders [1]. Therefore, understanding the factors associated with PE in the general population is crucial for implementing prevention strategies and developing effective interventions.

Childhood adversity (CA) has been identified as a significant transdiagnostic risk factor affecting mental health, from subtle symptoms to chronic and severe conditions [2]. Some researchers propose a dimensional model of CA, categorizing subtypes into two domains, each linked to unique biological processes. The threat domain includes subtypes such as sexual abuse (SA), physical abuse (PA), or emotional abuse (EA), while the deprivation domain refers to the absence of essential cognitive, social, or emotional input (i.e., emotional neglect (EN) and physical neglect (PN)) [3–5]. Furthermore, studies have shown a dose–response relationship between the number of childhood adversities and mental health outcomes [6]. Moreover, research has recently suggested that specific CA subtypes may have distinct impacts on mental health, especially when considering sex-specific effects [7–10].

Incorporating sex differences into research [11] and clinical care (e.g., early psychosis clinics) [12] is essential to improve mental health outcomes and reduce disparities. Emerging evidence indicates that sex differences shape the impact of CA subtypes on psychosis [9, 10]. Some studies suggest that especially SA and PA may have stronger associations with psychosis and mental health in females [9, 10, 13, 14]. A recent meta-analysis found a link between SA and psychosis in females but not in males [9]. Another meta-analysis reported stronger associations of PA and SA with anxiety or depression in females [14]. However, follow-up comparisons showed no significant sex differences. This may reflect the limited number of studies examining subtype-specific effects by sex. Additionally, many studies fail to account for the co-occurrence and interdependence of these subtypes.

Adding to its complexity, the impact of CA on mental health is influenced not only by the nature and severity of exposure but also by coping strategies. Researchers propose that coping strategies may be either adaptive or maladaptive, depending on the subjective experience, the contextual demands, and the resulting outcomes [15]. Notably, individuals at clinical high risk for psychosis tend to rely on maladaptive coping strategies [16], potentially worsening symptoms. Furthermore, sex differences in coping are evident. Females typically utilize a broader range of strategies, favoring social support and emotional expression [17–20], while males often adopt avoidant strategies [18]. Despite these insights, the sex-specific effects of coping strategies on PE remain insufficiently explored [17, 21, 22], particularly in the general population. Lin et al. reported that females use a broader range of coping strategies, but the pathways linking coping strategies to PE were similar across sexes [21]. Other studies examining the role of sex in the association between coping strategies and PE have focused on case–control comparisons, such as psychosis-prone groups versus healthy controls, without considering the dimensional conceptualization of the psychosis phenotype [23, 24]. Therefore, further research is needed.

This study aims to explore sex differences in how CA and coping strategies influence PE, and whether coping strategies moderate the relationship between CA and PE in the general population.

## Methods

### Participants

This study utilized data from the TwinssCan Project, a general population cohort that includes monozygotic and dizygotic twins, their siblings (15–35 years), and parents. Recruitment was conducted through the East Flanders Prospective Twin Survey (EFPTS), a population-based registry of multiple births in Belgium [25]. Detailed information on participant recruitment is available in previous reports [26]. All participants provided written informed consent, and parental consent was obtained for those under 18. Participants were excluded if caregivers reported a severe psychiatric condition impairing functioning and study participation. The local ethics committee (Commissie Medische Ethiek van de Universitaire Ziekenhuizen KU Leuven, Nr. B32220107766) approved the study. The current analyses included data from 790 participants, twins and their non-twin siblings, with relevant information.

### Measurements

#### Psychosis expression

PE was assessed using the Community Assessment of Psychic Experiences (CAPE), a validated 42-item self-report questionnaire [27]. CAPE is widely used for evaluating PE in the general population and has demonstrated strong reliability and validity [28]. It provides a total and three subdomains: positive, negative, and depressive. The questionnaire measures both the frequency of experiences and the distress associated with them, using a four-point Likert scale. Total and subscale mean scores range from 1 to 4. Consistent with previous studies using this cohort [29], the analyses focused on frequency scores.

#### Coping strategies

Coping strategies were assessed using the Utrecht Coping List (UCL), a 44-item self-report questionnaire designed to evaluate seven coping strategies [30, 31]. Participants rated each item on a four-point Likert scale ranging from 1 (never) to 4 (very often). Active coping (7 items) reflects proactive problem-solving strategies to address stressors. Seeking social support (6 items) measures the extent to which individuals turn to others for emotional or practical assistance. Reassuring thoughts (5 items) involve maintaining a positive mindset or reframing negative situations. Emotional expression (3 items) refers to openly displaying emotions such as anger or fear. Passive-reacting coping (7 items) captures feelings of being overwhelmed, helpless, and withdrawn in response to stress. Palliative-reacting coping (8 items) focuses on distraction-based behaviors, such as smoking or drinking, to alleviate distress. Avoidance (8 items) reflects the tendency to ignore or deny stressful situations rather than confronting them directly.

#### Childhood adversity

CA was assessed using the Childhood Trauma Questionnaire (CTQ), a 28-item self-report measure that evaluates adverse experiences before the age of 17 [32]. Each item was rated on a five-point Likert scale, covering five subtypes: physical abuse, emotional abuse, sexual abuse, physical neglect, and emotional neglect. To obtain a total CA score, responses to all items were summed. In line with the CTQ manual and following our consistent approach in previous studies [8, 33–35], subtypes were dichotomized based on predefined cut-off scores:≥9 for EA, ≥8 for PA, ≥6 for SA, ≥10 for EN, and ≥ 8 for PN.

### Statistical analyses

All statistical analyses were conducted using Stata version 18.0 [36]. To explore sex-specific associations, mutually adjusted stratified linear regression analyses were conducted to examine the association of PE with CA and coping strategies, which included all adversity subtypes and all coping strategies, respectively. The CAPE total score was the primary outcome, while the CAPE negative, positive, and depressive subscales were analyzed as secondary outcomes. Sensitivity analyses were conducted to assess the robustness of the findings by testing the associations between total CA, its subtypes, and the coping strategies in independent models. The explained variance (adjusted R^2^) for the independent regression models was also calculated. Additionally, exploratory interaction analyses investigated whether coping strategies moderated the association between CA and PE (total CAPE and its subdomains) in independent models. Post-hoc analyses examined sex differences in significant associations by comparing regression coefficients between males and females using Chow’s test [37]. In all models, to account for intrafamily correlation, standard errors were adjusted for sibling clustering using Stata’s “cluster” option. All analyses were adjusted for age. Statistical significance was corrected for multiple comparisons using Bonferroni correction (see the results section and Supplementary Table 1).

## Results

Sample characteristics are reported in Table 1, and missing data are reported in Supplementary Table 2. After excluding participants with missing data in any variables, the final sample consisted of 790 twins and siblings (312 males, 478 females). The mean age was 17.45 ± 3.58 years.

**Table 1.** Sample characteristics

	Males (*n* = 312)	Females (*n* = 478)
**Age (years), M (SD)**	16.89 (±2.76)	17.82 (±3.98)
**Zygosity, *n* (%)**		
MZ	98 (31.41%)	176 (36.82%)
DZ	201 (64.42%)	272 (56.90%)
Sibling	13 (4.17%)	30 (6.28%)
**Psychosis expression M (SD)**		
Total CAPE	1.56 (±0.26)	1.57 (±0.27)
Positive subdomain	1.42 (±0.28)	1.41 (±0.28)
Negative subdomain	1.72 (±0.36)	1.65 (±0.34)
Depressive subdomain	1.67 (±0.34)	1.82 (±0.37)
**Coping strategies, M (SD)**		
Active coping	17.87 (±3.48)	16.99 (±3.14)
Seeking social support	12.83 (±3.10)	15.42 (±3.84)
Reassuring thoughts	12.23 (±2.55)	12.72 (±2.50)
Emotional expression	6.86 (±1.77)	6.78 (±1.78)
Passive-reacting coping	11.80 (±3.17)	12.62 (±3.53)
Palliative-reacting coping	18.23 (±3.07)	18.32 (±3.57)
Avoidance	16.88 (±3.45)	16.45 (±3.34)
**Total CA, M (SD)**	34.69 (±8.17)	33.81 (±8.84)
**CA subtypes, *n* (%)**		
Emotional abuse	101 (32.37%)	146 (30.54%)
Physical abuse	20 (6.41%)	14 (2.93%)
Sexual abuse	18 (5.77%)	37 (7.74%)
Emotional neglect	163 (52.24%)	177 (37.03%)
Physical neglect	54 (17.31%)	74 (15.48%)

DZ, dizygotic; MZ, monozygotic.

*Note:* Means (M) and standard deviations (SD) are reported for continuous variables. For categorical variables, counts (*n*) and percentages (%) are provided.

### Childhood adversity and psychosis expression

#### Association between CA and CAPE total

**Figure 1.**
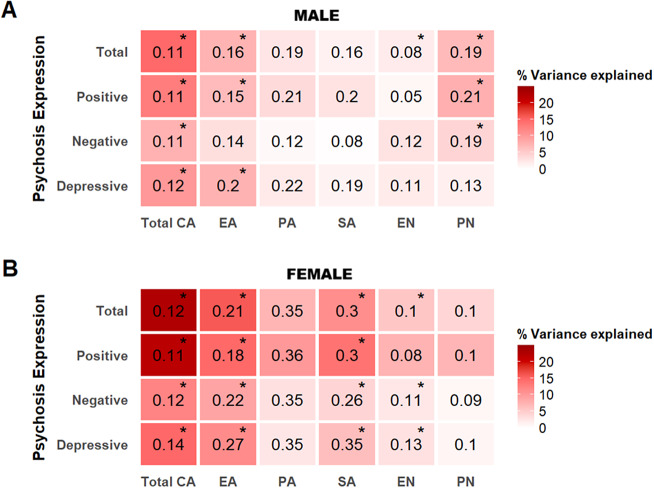
Sex-stratified variance in psychosis expression explained by childhood adversity subtypes. The heatmaps show the variance in psychosis expression explained by each childhood adversity subtype in males (A) and females (B) from the independent models. Unstandardized regression coefficients (Beta) are shown, with significant associations marked by asterisks. All models were adjusted for age. EA, emotional abuse; EN, emotional neglect; PN, physical neglect; PA, physical abuse; SA, sexual abuse.

In males, EA and PN were significantly associated with increased CAPE total score in the mutually adjusted model (*p* < 0.05; Table 2). Independent regression analyses confirmed these associations and additionally showed significant associations of total CA and EN with CAPE total scores (Bonferroni corrected *p* < 0.05/6). The model including EA had the highest *R* ^2^ (7.5%) among the adversity subtypes (Figure 1A).

**Table 2.** Main effects of childhood adversity and coping strategies on CAPE total in mutual models

	Males (*n* = 312)			Females (*n* = 478)			Sex Differences	
	Beta	95% CI	*p*-value	Beta	95% CI	*p*-value	χ^2^	*p*-value
**Childhood adversity^a^**								
Emotional abuse	0.11	0.05 to 0.17	**<0.001**	0.16	0.11 to 0.21	**<0.001**	1.75	0.19
Physical abuse	0.05	−0.06 to 0.17	0.368	0.20	0.07 to 0.33	**0.003**	0.60	0.44
Sexual abuse	0.02	−0.11 to 0.15	0.749	0.18	0.11 to 0.26	**<0.001**	3.40	0.07
Emotional neglect	0.03	−0.02 to 0.09	0.227	0.02	−0.03 to 0.07	0.483	N/A	N/A
Physical neglect	0.12	0.04 to 0.19	**0.002**	0.00	−0.06 to 0.06	1.000	6.12	**0.01**
**Coping strategies^b^**								
Active coping	−0.017	−0.044 to 0.010	0.221	−0.002	−0.024 to 0.021	0.891	N/A	N/A
Seeking social support	0.014	−0.017 to 0.046	0.378	−0.051	−0.072 to −0.031	**<0.001**	11.0	**0.001**
Reassuring thoughts	0.017	−0.012 to 0.047	0.250	−0.013	−0.036 to 0.010	0.285	N/A	N/A
Emotional expression	−0.022	−0.049 to 0.004	0.101	0.028	0.007 to 0.048	**0.008**	7.62	**0.006**
Passive-reacting	0.140	0.111 to 0.170	**<0.001**	0.125	0.105 to 0.146	**<0.001**	0.35	0.552
Palliative-reacting	−0.015	−0.047 to 0.017	0.357	0.001	−0.021 to 0.023	0.945	N/A	N/A
Avoidance	0.016	−0.012 to 0.043	0.274	0.017	−0.005 to 0.039	0.127	N/A	N/A

a *p* Value threshold was accepted as < 0.05 for one mutually adjusted model including all childhood adversity subtypes on CAPE total.

b *p* Value threshold was accepted as < 0.05 for one mutually adjusted model including all coping strategies on CAPE total. p < 0.05 was accepted as significance level.

In females, EA, PA, and SA were significantly associated with increased CAPE total score in the mutually adjusted model (*p* < 0.05; Table 2). Independent regression analyses confirmed the associations with EA and SA and additionally showed significant associations of total CA and EN with the CAPE total score (*p* < 0.05/6). The model including EA had the highest *R* ^2^ (15.8%) among the adversity subtypes (Figure 1B).

Follow-up sex comparisons (*p* < 0.05) using coefficients from the mutually adjusted model indicated an association between PN and CAPE total specifically in males (χ^2^ = 6.12, *p* = 0.01; Table 2).

#### Association between CA and CAPE subdomains

In males, PN was significantly associated with increased scores in the positive and negative subdomains, while EA was significantly associated with increased scores in the positive and depressive subdomains in the mutually adjusted models (*p* < 0.05/3; Supplementary Table 4). These associations were confirmed in the independent models (*p* < 0.05/18; Supplementary Table 5, Figure 1A). Total CA was associated with higher scores across all CAPE subdomains, with the model including the positive subdomain having the highest *R* ^2^ (12.8%; Figure 1A).

In females, EA and SA were significantly associated with increased scores across all subdomains in the mutually adjusted models (*p* < 0.05/3; Supplementary Table 4). These associations were confirmed in the independent models (*p* < 0.05/18; Supplementary Table 5, Figure 1B). Additionally, EN was associated with higher scores in the negative and depressive subdomains. Total CA was associated with higher scores across all CAPE subdomains, with the model including the positive subdomain having the highest *R* ^2^ (21.8%; Figure 1B).

Follow-up sex comparisons (*p* < 0.05) using coefficients from the mutually adjusted model indicated associations between PN and the positive and negative subdomains, specifically in males (Supplementary Table 4).

### Coping strategies and psychosis expression

#### Association between coping strategies and CAPE total

**Figure 2.**
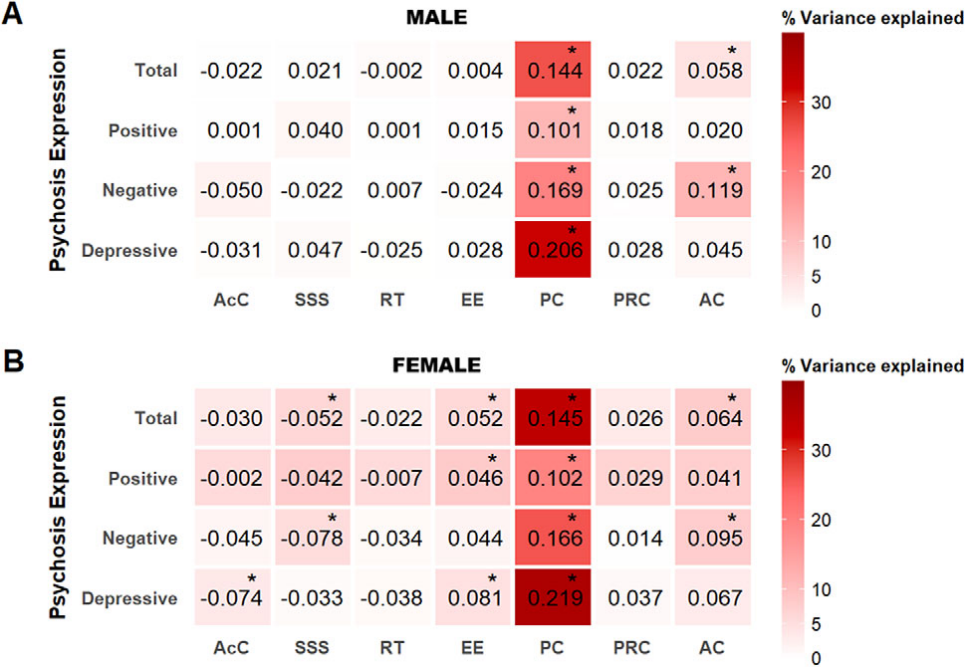
Sex-stratified variance in psychosis expression explained by coping strategies. The heatmaps show the variance in psychosis expression explained by each coping strategy in males (A) and females (B) from the independent models. Unstandardized regression coefficients (Beta) are shown, with significant associations marked by asterisks. All models were adjusted for age. AcC, active coping; AC, avoidance coping; EE, emotional expression; PC, passive-reacting coping; PRC, palliative-reacting coping; RT, reassuring thoughts; SSS, seeking social support.

In males, passive-reacting was significantly associated with increased CAPE total in the mutually adjusted model (*p* < 0.05; Table 2). The association was confirmed in the independent models, which additionally showed that avoidance was significantly associated with increased CAPE total. The model with passive-reacting had the highest *R* ^2^ (*p* < 0.05/7; 26.1%; Figure 2A).

In females, seeking social support was significantly associated with decreased CAPE total scores, whereas passive-reacting and emotional expression were associated with increased CAPE total scores (*p* < 0.05; Table 2). The associations were confirmed in the independent models (*p* < 0.05/7), which additionally showed that avoidance was significantly associated with increased CAPE total. The model with passive-reacting had the highest *R* ^2^ (33.9%; Figure 2B).

Follow-up sex comparisons (*p* < 0.05) using coefficients from the mutually adjusted model indicated female-specific associations between CAPE total and seeking social support as well as emotional expression (Table 2).

#### Association between coping strategies and CAPE subdomains

In males, passive-reacting was associated with higher CAPE scores across all subdomains, while avoidance and emotional expression were associated with higher and lower scores in the negative subdomain, respectively (*p* < 0.05/3; Supplementary Table 6). The independent models confirmed these associations, except for the association with emotional expression, which was not significant (*p* < 0.05/21; Supplementary Table 7). The model including passive-reacting on the depressive domain had the highest *R* ^2^ (32.1%; Figure 2A).

In females, passive-reacting was associated with higher CAPE scores across all subdomains in the mutually adjusted models, and avoidance with higher scores in the negative subdomain. Seeking social support was associated with lower scores in the positive and negative subdomains, while active coping was associated with lower scores in the depressive subdomain (*p* < 0.05/3; Supplementary Table 6). In the independent models, these associations were confirmed, except for social support in the positive subdomain, which was not significant. In addition, emotional expression was associated with higher scores in the positive and depressive subdomains (*p* < 0.05/21; Supplementary Table 7). The model including passive-reacting on the depressive subdomain had the highest *R* ^2^ (36.9%; Figure 2B).

Follow-up sex comparisons (*p* < 0.05) using coefficients from the mutually adjusted model indicated that seeking social support was associated with lower positive and negative subdomain scores specifically in females, whereas emotional expression was associated with lower negative subdomain scores specifically in males (Supplementary Table 6).

### Exploratory interaction analyses between coping strategies and CA on psychosis expression

#### Interaction effects on CAPE total

In males, interactions including avoidance (× total CA/SA/PA) and passive-reacting (× PN) were associated with increased CAPE total scores, whereas the interaction including reassuring thoughts (× EN) was associated with decreased CAPE total scores (Supplementary Table 8).

In females, interactions including seeking social support (× total CA/EA/PA/PN), reassuring thoughts (× total CA/PA/EN/PN) and palliative-reacting (× total CA/PA/PN) were associated with decreased total CAPE scores. In contrast, passive-reacting interacted with total CA and EN, which was associated with increased total CAPE scores (Supplementary Table 8).

**Figure 3.**
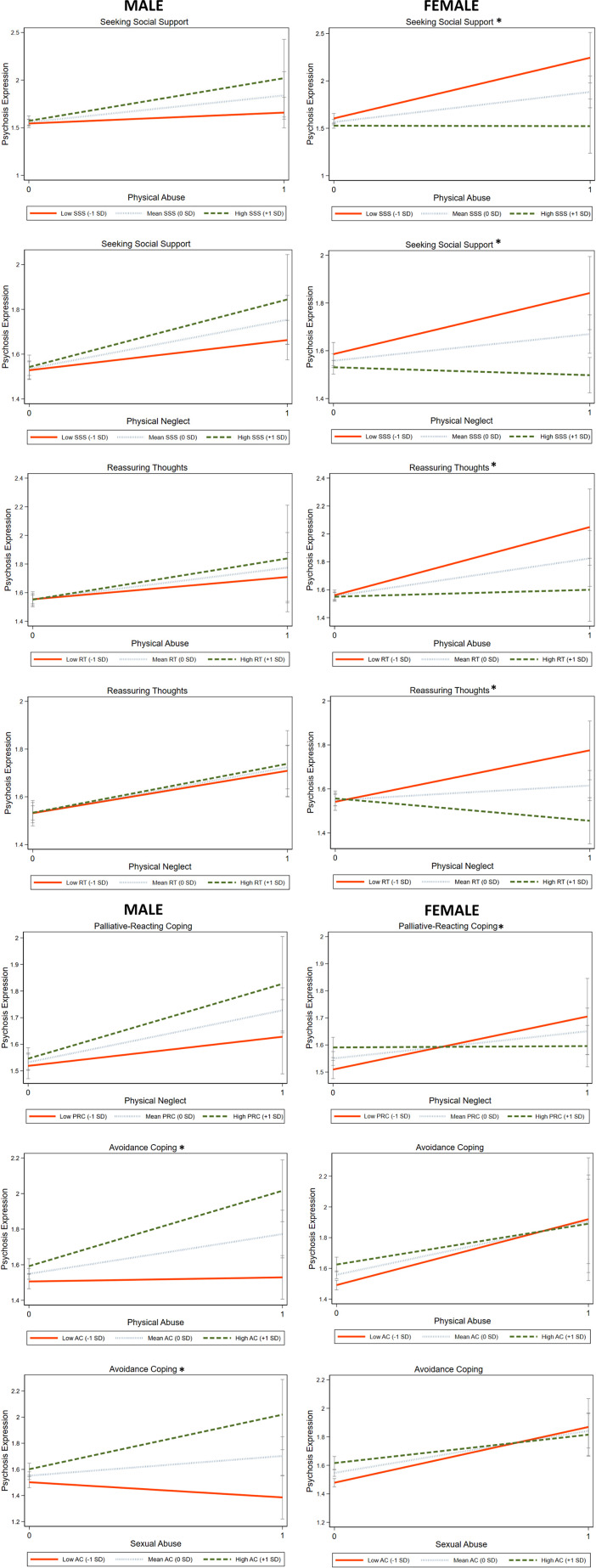
Interaction effects of CA subtypes and coping strategies on psychosis expression. The figure illustrates the interaction effects between adversity subtypes and coping strategies on total CAPE that were significantly different in males and females (*indicates the significant model). Sex-stratified margin plots show the linear prediction of total CAPE frequency scores at specified values of childhood adversity subtypes and coping strategies, including seeking social support, reassuring thoughts, palliative-reacting, and avoidance, while keeping age constant at the mean value. AC, avoidance coping; CAPE, community assessment of psychic experiences; PRC, palliative-reacting coping; RT, reassuring thoughts; SD, standard deviation; SSS: seeking social support.

Follow-up sex comparisons revealed significant differences. Specifically, in females, seeking social support and reassuring thoughts weakened the effects of both PA and PN, while palliative-reacting coping weakened the effect of PN on total CAPE scores. In males, avoidance strengthened the effects of SA and PA on total CAPE scores (Figure 3, Supplementary Table 8).

#### Interaction effects on CAPE subdomains

For CAPE subdomains, interaction patterns largely mirrored those observed for CAPE total scores, with some additional subdomain-specific effects (Supplementary Tables 9–11).

Follow-up sex comparisons indicated several significant differences. Seeking social support (× total CA/PA/PN), reassuring thoughts (× PA/PN), and palliative-reacting (× PN) were associated with lower subdomain scores, whereas emotional expression (× SA) was associated with higher positive scores particularly in females. Avoidance (× SA/PA) was associated with higher negative and depressive scores particularly in males. Notably, avoidance showed a sex-divergent pattern in the context of SA, being significantly associated with lower scores in females (Supplementary Tables 9–11).

## Discussion

This study examines sex-specific associations of PE with CA and coping strategies in the general population. CA was linked to PE in both sexes, with notable differences for specific subtypes. In males, EA and PN showed significant associations with PE, whereas in females, EA, PA, and SA were associated with PE. Coping strategies also differed. Passive-reacting was associated with increased PE in both sexes. Additionally, seeking social support and emotional expression were linked to decreased and increased PE, respectively, in females. Furthermore, interaction analyses indicated that these associations were not uniform but depended on the combination of adversity type, coping strategy, and sex.

Total CA was significantly associated with CAPE total and subdomain scores in both sexes, supporting prior research identifying CA as an important environmental risk factor for psychosis [2, 9]. EA emerged as a trans-syndromal risk factor across sexes, explaining the highest variance in total CAPE scores among the CA subtypes, consistent with prior studies that show its broad impact [38, 39]. Sex-specific patterns also emerged. The association between PN and PE in males may reflect heightened sensitivity to deprivation-related stressors [8], aligning with prior findings on its specific impact in males [40] and sex-stratified research linking it to negative symptoms in males [13, 38, 41]. The unique association between SA and all the PE dimensions in females aligns with evidence suggesting greater vulnerability to abuse-related adversity [9, 10, 13, 14, 42–44]. Neurodevelopmental evidence supports these findings, showing sex-specific effects of early adversity on brain development; for example, hippocampal volume relates to neglect in males and abuse in females at different developmental stages [45]. Our findings underscore the complexity of adversity’s impact, as exposures are often interdependent and interconnected [46]. Future research should replicate these findings in larger cohorts and explore additional adversities that may exhibit sex-specific effects [47].

Coping strategies play a crucial role in linking adversity to mental health outcomes. Consistent with previous research, females in our sample utilized a broader range of coping strategies [21]. Although a prior study did not identify sex-specific pathways between coping strategies and PE in the general population [21], we observed both shared and sex-specific associations. Passive-reacting consistently emerged as a maladaptive strategy. This finding is generally in line with previous research indicating that maladaptive coping contributes to psychosis across sexes [48–50]. Passive-reacting has been shown to be associated with an increased impact of stressful life events and poorer clinical outcomes, particularly in individuals with psychosis spectrum disorders and their unaffected relatives [51, 52]. Sex-specific associations with coping styles also emerged. The association between seeking social support and reduced psychosis expression in females is consistent with prior literature [18, 53–55], highlighting its role in emotional regulation and stress reduction [56]. Conversely, while previous research indicates that males have a greater tendency toward emotional suppression, emotional expression could be beneficial [57, 58].

Interaction analyses of coping strategies and CA on PE underscored further sex differences. The use and effect of some coping strategies depended on the specific stressor [19]. Females’ broader repertoire [19], including seeking social support, palliative-reacting, and reassuring thoughts, was generally protective, while passive-reacting was maladaptive. Notably, avoidance showed associations with lower levels of depressive and negative symptoms among females when interacting with SA, suggesting that in some contexts it may provide temporary relief from distressing thoughts or emotions [59]. This interpretation is consistent with evidence that females report higher levels of rumination [60] and that rumination may partly mediate the association between childhood adversity and affective symptoms [61]. In contrast, and consistent with prior research [62, 63], avoidance was associated with poorer outcomes in males, especially in interaction with PA or SA. In males, avoidance may reflect emotional suppression and social disengagement, contributing to less favorable outcomes. Psychosocial perspectives, such as social role theories of gender differences, further emphasize women’s greater reliance on social support [64], and men’s tendency toward emotional suppression and avoidance [65]. Conversely, reassuring thoughts was beneficial for males, suggesting potential pathways for resilience, particularly against EN. Clinically, these insights underscore both the broad utility of trauma-focused interventions across sexes and the need for sex-sensitive approaches tailored to distinct coping tendencies.

### Limitations

Although this study provides important insights into sex differences, several limitations should be noted. The relatively small sample size may have limited statistical power, particularly in stratified analyses, reducing sensitivity to subtle effects. The relatively young mean age of the sample corresponds to a developmental period in which subclinical PE is common [66], and may represent an early transdiagnostic marker for later psychopathology [67]. While this enhances the developmental relevance of the findings, it may also limit their generalizability to older populations, underscoring the need for replication across broader age ranges. Post-hoc analyses did not consistently confirm sex differences observed in stratified analyses, consistent with prior meta-analyses, suggesting these effects may be modest. However, small effects may still be meaningful within the broader exposome framework, as they can accumulate and interact with biological, psychological, and social factors over time. Larger studies incorporating additional factors are needed. Including twins and siblings increased statistical power but may have introduced bias due to shared genetic and environmental influences; to address this, intrafamily correlation was accounted for and standard errors were adjusted for sibling clustering. Prior literature suggests that including all siblings, rather than one per pair, improves statistical power [68]. While the mutually adjusted models accounted for other childhood adversity subtypes, individual coefficients should be interpreted with caution, as confounding by unmeasured factors cannot be fully excluded. Finally, the cross-sectional design precludes causal inferences, as coping strategies may both shape and be shaped by PE [21], and could mediate the association between adversity and psychosis-related outcomes [49, 69], as well as the link between PE and later functioning [50]. Longitudinal studies are essential to clarify these dynamics.

## Conclusion

This study shows that CA and coping strategies are associated with PE through both shared and sex-specific patterns. CA, particularly EA, emerged as a trans-syndromal risk factor in both sexes, while other subtypes showed sex-specific associations, with SA in females and PN in males. Coping further shaped these relationships, with passive-reacting explaining the greatest variance across sexes. Males more often relied on maladaptive strategies, whereas females particularly benefited from supportive approaches such as seeking social support. Overall, coping effects varied by adversity type and sex, underscoring the need to move beyond one-size-fits-all care models. Integrating sex-sensitive frameworks that consider adversity histories and coping profiles may improve early intervention strategies.

## Supporting information

10.1192/j.eurpsy.2026.10173.sm001 Karaçam Doğan et al. supplementary material Karaçam Doğan et al. supplementary material

## Data Availability

Data are available upon reasonable request.

## Supplementary material

The supplementary material for this article can be found at http://doi.org/10.1192/j.eurpsy.2026.10173.
