# Supplementary material for: Sex differences in the effect of childhood adversity and coping strategies on psychosis expression: A TwinssCan study
Source: Eur Psychiatry. 2026 Feb 20;69(1):e42. doi: 10.1192/j.eurpsy.2026.10173 (PMC13122524; doi:10.1192/j.eurpsy.2026.10173)
Supplement: Karaçam Doğan et al. supplementary material [file S0924933826101734sup001.docx]

Supplementary Table 1- Bonferroni corrections

| **Analyses** | **Explanation** | **p-value** |
| --- | --- | --- |
| **Main analyses** on CAPE total |  |  |
| Childhood adversity | 1 mutually adjusted model including all childhood adversity subtypes on CAPE total | <0.05 |
| Coping strategies | 1 mutually adjusted model including all coping strategies on CAPE total | <0.05 |
| **Secondary analyses** on CAPE subdomains |  |  |
| Childhood adversity | 3 mutually adjusted models including all childhood adversity subtypes on 3 CAPE subdomains | <0.016 |
| Coping strategies | 3 mutually adjusted models including all coping styles on 3 CAPE subdomains | <0.016 |
| **Sensitivity analyses** on CAPE total |  |  |
| Childhood adversity | 6 independent models for total childhood adversity and its subtypes on CAPE total | <0.0083 |
| Coping strategies | 7 independent models for coping styles on CAPE total | <0.0071 |
| **Sensitivity analyses** on CAPE subdomains |  |  |
| Childhood adversity | 18 independent models for 6 total childhood adversity and its subtypes on 3 CAPE subdomains | <0.0027 |
| Coping strategies | 21 independent models for 7 coping styles on 3 CAPE subdomains | <0.0023 |
| **Explorative analyses** | Bonferroni correction was not applied for the interaction analyses | <0.05 |
| **Follow-up comparisons** | Bonferroni correction was not applied | <0.05 |

| **Variables** | **Number of missing reports** | |
| --- | --- | --- |
|  | **Males** | **Females** |
| **Age** | 0 | 0 |
| **Psychosis expression** |  |  |
| Total CAPE score | 15 | 11 |
| Positive subscore | 15 | 11 |
| Negative subscore | 15 | 11 |
| Depressive subscore | 15 | 11 |
| **Coping strategies** |  |  |
| Active coping | 15 | 7 |
| Seeking social support | 15 | 7 |
| Reassuring thoughts | 15 | 7 |
| Emotional expression | 15 | 7 |
| Passive-reacting coping | 15 | 7 |
| Palliative-reacting coping | 15 | 7 |
| Avoidance coping | 15 | 7 |
| **Childhood adversity** |  |  |
| Total CA | 15 | 10 |
| Emotional abuse | 15 | 10 |
| Physical abuse | 14 | 10 |
| Sexual abuse | 15 | 10 |
| Emotional neglect | 15 | 10 |
| Physical neglect | 15 | 10 |

Supplementary Table 2- The number of missing reports in males and females

| Supplementary Table 3- Sensitivity analyses of childhood adversity and coping strategies on CAPE total in independent models | | | | | | | | | | |
| --- | --- | --- | --- | --- | --- | --- | --- | --- | --- | --- |
|  | **Males (n=312)** | | | | **Females (n=478)** | | | | **Sex Differences** | |
|  | Beta | 95% CI | *p*-value | Adjusted R^2*^ | Beta | 95% CI | *p*-value | Adjusted R^2*^ | χ² | *p*-value |
| **Childhood adversity^a^** |  |  |  |  |  |  |  |  |  |  |
| Total CA | 0.11 | 0.07 to 0.15 | **<0.001** | 14.7% | 0.12 | 0.09 to 0.15 | **<0.001** | 22.7% | 0.13 | 0.71 |
| Emotional abuse | 0.16 | 0.09 to 0.22 | **<0.001** | 7.5% | 0.21 | 0.15 to 0.27 | **<0.001** | 15.8% | 1.55 | 0.21 |
| Physical abuse | 0.19 | -0.01 to 0.39 | 0.069 | 2.5% | 0.35 | 0.08 to 0.62 | 0.011 | 7.3% | N/A | N/A |
| Sexual abuse | 0.16 | -0.05 to 0.37 | 0.140 | 1.3% | 0.30 | 0.18 to 0.41 | **<0.001** | 11.2% | 1.26 | 0.26 |
| Emotional neglect | 0.08 | 0.02 to 0.14 | **0.008** | 1.9% | 0.10 | 0.04 to 0.15 | **0.001** | 5.6% | 0.15 | 0.70 |
| Physical neglect | 0.19 | 0.10 to 0.28 | **<0.001** | 6.8% | 0.10 | 0.01 to 0.18 | 0.029 | 4.1% | 2.03 | 0.15 |
| **Coping strategies^b^** |  |  |  |  |  |  |  |  |  |  |
| Active coping | -0.02 | -0.05 to 0.01 | 0.137 | 0.2% | -0.03 | -0.06 to -0.001 | 0.042 | 3.6% | N/A | N/A |
| Seeking social support | 0.02 | -0.01 to 0.06 | 0.247 | -0.2%** | -0.05 | -0.08 to -0.02 | **0.001** | 6.3% | 9.82 | **0.002** |
| Reassuring thoughts | -0.002 | -0.04 to 0.03 | 0.896 | -0.6%** | -0.02 | -0.05 to 0.01 | 0.157 | 3.1% | N/A | N/A |
| Expressing emotions | 0.004 | -0.03 to 0.04 | 0.785 | -0.5%** | 0.05 | 0.02 to 0.08 | **0.001** | 6.1% | 4.60 | **0.03** |
| Passive-reacting coping | 0.14 | 0.11 to 0.18 | **<0.001** | 26.1% | 0.15 | 0.12 to 0.17 | **<0.001** | 33.9% | 0.01 | 0.94 |
| Palliative-reacting coping | 0.02 | -0.01 to 0.06 | 0.218 | -0.04%** | 0.03 | 0.001 to 0.05 | 0.039 | 3.4% | N/A | N/A |
| Avoidance | 0.06 | 0.02 to 0.09 | **0.001** | 4.5% | 0.06 | 0.03 to 0.10 | **<0.001** | 7.9% | 0.07 | 0.80 |

Comparison analyses were conducted only when stratified analyses revealed significant results (after Bonferroni correction) in either males or females, or both. The models were adjusted for:

**^a^** Bonferroni correction applied for 6 models examining total childhood adversity (CA) and its subtypes (p<0.0083).

**^b^** Bonferroni correction applied for 7 models testing different coping strategies (p<0.0071).

*Adjusted R^2^ values are presented to demonstrate the proportion of variance explained by each respective model including adjustments for age and family relatedness.

** Adjusted R² values were negative, indicating that the included variables explained less variance in psychosis expression than the mean-only model.

Supplementary Table 4- Secondary analyses of childhood adversity on CAPE subdomains in mutually adjusted models

|  | **Males** | | | **Females** | | | | **Sex Differences** | |
| --- | --- | --- | --- | --- | --- | --- | --- | --- | --- |
|  | Beta | 95% CI | *p*-value* | Beta | 95% CI | | *p*-value* | χ² | *p*-value |
| **CAPE positive** | | | | | | | | | |
| Emotional abuse | 0.11 | 0.03 to 0.19 | **0.005** | 0.14 | 0.09 to 0.20 | **<0.001** | | 0.49 | 0.49 |
| Physical abuse | 0.09 | -0.07 to 0.25 | 0.276 | 0.19 | -0.06 to 0.44 | 0.132 | | N/A | N/A |
| Sexual abuse | 0.05 | -0.12 to 0.23 | 0.545 | 0.23 | 0.12 to 0.33 | **<0.001** | | 2.57 | 0.11 |
| Emotional neglect | -0.01 | -0.08 to 0.05 | 0.669 | 0.0002 | -0.05 to 0.05 | 0.994 | | N/A | N/A |
| Physical neglect | 0.16 | 0.07 to 0.26 | **0.001** | 0.02 | -0.05 to 0.09 | 0.571 | | 5.78 | **0.02** |
| **CAPE negative** | | | | | | | | | |
| Emotional abuse | 0.09 | 0.001 to 0.19 | 0.046 | 0.18 | 0.10 to 0.26 | **<0.001** | | 1.85 | 0.17 |
| Physical abuse | 0.02 | -0.21 to 0.25 | 0.838 | 0.19 | -0.09 to 0.47 | 0.179 | | N/A | N/A |
| Sexual abuse | -0.06 | -0.30 to 0.18 | 0.625 | 0.17 | 0.04 to 0.30 | **0.008** | | 2.92 | 0.09 |
| Emotional neglect | 0.07 | -0.01 to 0.16 | 0.080 | 0.02 | -0.05 to 0.09 | 0.497 | | N/A | N/A |
| Physical neglect | 0.16 | 0.05 to 0.26 | **0.004** | -0.01 | -0.10 to 0.08 | 0.825 | | 5.57 | **0.02** |
| **CAPE depressive** | | | | | | | | | |
| Emotional abuse | 0.16 | 0.07 to 0.25 | **<0.001** | 0.22 | 0.15 to 0.30 | **<0.001** | | 1.16 | 0.28 |
| Physical abuse | 0.09 | -0.15 to 0.32 | 0.467 | 0.13 | -0.14 to 0.40 | 0.342 | | N/A | N/A |
| Sexual abuse | 0.04 | -0.21 to 0.28 | 0.775 | 0.26 | 0.12 to 0.40 | **<0.001** | | 2.54 | 0.11 |
| Emotional neglect | 0.05 | -0.03 to 0.12 | 0.240 | 0.03 | -0.04 to 0.10 | 0.391 | | N/A | N/A |
| Physical neglect | 0.06 | -0.06 to 0.18 | 0.322 | -0.01 | -0.11 to 0.09 | 0.858 | | N/A | N/A |

* p value threshold was accepted as <0.016 for 3 mutually adjusted models including all childhood adversity subtypes on 3 CAPE subdomains

|  | **Males** | | | | **Females** | | | | **Sex Differences** | |
| --- | --- | --- | --- | --- | --- | --- | --- | --- | --- | --- |
|  | Beta | 95% CI | *p*-value**^a^** | Adjusted R^2^**^b^** | Beta | 95% CI | *p*-value**^a^** | Adjusted R^2^**^b^** | χ² | *p*-value |
| **CAPE positive** | | | | | | | | | | |
| Total CA | 0.11 | 0.07 to 0.15 | **<0.001** | 12.8% | 0.11 | 0.07 to 0.15 | **<0.001** | 21.8% | 0.02 | 0.89 |
| Emotional abuse | 0.15 | 0.08 to 0.23 | **<0.001** | 6.7% | 0.18 | 0.12 to 0.25 | **<0.001** | 14.7% | 0.39 | 0.53 |
| Physical abuse | 0.21 | 0.01 to 0.42 | 0.038 | 3.6% | 0.36 | 0.06 to 0.65 | 0.019 | 10.1% | N/A | N/A |
| Sexual abuse | 0.20 | -0.02 to 0.42 | 0.072 | 2.9% | 0.30 | 0.17 to 0.43 | **<0.001** | 13.8% | 0.58 | 0.45 |
| Emotional neglect | 0.05 | -0.02 to 0.11 | 0.140 | 0.9% | 0.08 | 0.02 to 0.14 | 0.006 | 7.5% | N/A | N/A |
| Physical neglect | 0.21 | 0.10 to 0.31 | **<0.001** | 7.8% | 0.10 | 0.01 to 0.18 | 0.022 | 7.2% | 2.53 | 0.11 |
| **CAPE negative** | | | | | | | | | | |
| Total CA | 0.11 | 0.05 to 0.16 | **<0.001** | 7.6% | 0.12 | 0.08 to 0.15 | **<0.001** | 12.1% | 0.1 | 0.76 |
| Emotional abuse | 0.14 | 0.05 to 0.23 | 0.003 | 3.2% | 0.22 | 0.14 to 0.29 | **<0.001** | 8.9% | 1.91 | 0.17 |
| Physical abuse | 0.12 | -0.11 to 0.36 | 0.289 | 0.7% | 0.35 | 0.05 to 0.65 | 0.022 | 3.0% | N/A | N/A |
| Sexual abuse | 0.08 | -0.15 to 0.30 | 0.509 | 0.2% | 0.26 | 0.12 to 0.40 | **<0.001** | 4.2% | 1.81 | 0.18 |
| Emotional neglect | 0.12 | 0.03 to 0.20 | 0.007 | 2.7% | 0.11 | 0.04 to 0.18 | **0.0026** | 2.4% | 0.04 | 0.84 |
| Physical neglect | 0.19 | 0.09 to 0.30 | **<0.001** | 4.2% | 0.09 | -0.02 to 0.19 | 0.106 | 0.9% | 1.99 | 0.16 |
| **CAPE depressive** | | | | | | | | | | |
| Total CA | 0.12 | 0.06 to 0.18 | **<0.001** | 10.1% | 0.14 | 0.10 to 0.18 | **<0.001** | 14.8% | 0.41 | 0.52 |
| Emotional abuse | 0.20 | 0.12 to 0.29 | **<0.001** | 7.5% | 0.27 | 0.19 to 0.36 | **<0.001** | 11.4% | 1.38 | 0.24 |
| Physical abuse | 0.22 | -0.01 to 0.46 | 0.064 | 2.2% | 0.35 | 0.06 to 0.63 | 0.017 | 2.5% | N/A | N/A |
| Sexual abuse | 0.19 | -0.03 to 0.41 | 0.092 | 1.3% | 0.35 | 0.20 to 0.50 | **<0.001** | 6.4% | 1.38 | 0.24 |
| Emotional neglect | 0.11 | 0.03 to 0.18 | 0.006 | 2.0% | 0.13 | 0.05 to 0.21 | **<0.001** | 2.9% | 0.23 | 0.63 |
| Physical neglect | 0.13 | 0.01 to 0.25 | 0.040 | 1.6% | 0.10 | -0.01 to 0.21 | 0.065 | 1.0% | N/A | N/A |

Supplementary Table 5 – Sensitivity analyses of childhood adversity on CAPE subdomains in independent models

**^a^**p value threshold was accepted as <0.0027 for 18 models examining total childhood adversity and its subtypes

**^b^**Adjusted R^2^ values are presented to demonstrate the proportion of variance explained by each respective model including adjustments for age and family relatedness.

|  | **Males** | | | **Females** | | | | **Sex Differences** | |
| --- | --- | --- | --- | --- | --- | --- | --- | --- | --- |
|  | Beta | 95% CI | *p*-value* | Beta | 95% CI | | *p*-value* | χ² | *p*-value |
| **CAPE positive** | | | | | | | | | |
| Active coping | 0.002 | -0.032 to 0.036 | 0.902 | 0.021 | -0.009 to 0.051 | 0.168 | | N/A | N/A |
| Seeking social support | 0.029 | -0.011 to 0.068 | 0.152 | -0.056 | -0.085 to -0.027 | **<0.001** | | 12.31 | **<0.001** |
| Reassuring thoughts | 0.008 | -0.029 to 0.046 | 0.668 | -0.011 | -0.046 to 0.023 | 0.519 | | N/A | N/A |
| Emotional expression | -0.010 | -0.044 to 0.023 | 0.536 | 0.031 | 0.005 to 0.057 | 0.022 | | N/A | N/A |
| Passive-reacting coping | 0.106 | 0.056 to 0.157 | **<0.001** | 0.090 | 0.054 to 0.126 | **<0.001** | | 0.604 | 0.60 |
| Palliative-reacting coping | -0.008 | -0.045 to 0.029 | 0.674 | 0.016 | -0.012 to 0.043 | 0.259 | | N/A | N/A |
| Avoidance coping | -0.009 | -0.042 to 0.023 | 0.571 | -0.001 | -0.032 to 0.030 | 0.949 | | N/A | N/A |
| **CAPE negative** | | | | | | | | | |
| Active coping | -0.044 | -0.084 to -0.004 | 0.029 | -0.002 | -0.033 to 0.028 | 0.873 | | N/A | N/A |
| Seeking social support | -0.015 | -0.062 to 0.031 | 0.509 | -0.070 | -0.097 to -0.043 | **<0.001** | | 3.948 | **0.047** |
| Reassuring thoughts | 0.033 | -0.006 to 0.072 | 0.097 | -0.021 | -0.053 to 0.011 | 0.195 | | N/A | N/A |
| Emotional expression | -0.050 | -0.087 to -0.013 | **0.009** | 0.025 | -0.006 to 0.056 | 0.117 | | 9.086 | **0.003** |
| Passive-reacting coping | 0.159 | 0.117 to 0.202 | **<0.001** | 0.143 | 0.114 to 0.172 | **<0.001** | | 0.403 | 0.53 |
| Palliative-reacting coping | -0.026 | -0.075 to 0.024 | 0.314 | -0.018 | -0.047 to 0.011 | 0.227 | | N/A | N/A |
| Avoidance coping | 0.076 | 0.030 to 0.122 | **0.001** | 0.052 | 0.019 to 0.085 | **0.002** | | 0.731 | 0.39 |
| **CAPE depressive** | | | | | | | | | |
| Active coping | -0.019 | -0.056 to 0.018 | 0.309 | -0.045 | -0.078 to -0.011 | **0.009** | | 1.096 | 0.295 |
| Seeking social support | 0.031 | -0.007 to 0.070 | 0.112 | -0.024 | -0.057 to 0.009 | 0.158 | | N/A | N/A |
| Reassuring thoughts | 0.001 | -0.040 to 0.041 | 0.978 | -0.012 | -0.046 to 0.021 | 0.463 | | N/A | N/A |
| Emotional expression | -0.012 | -0.055 to 0.031 | 0.577 | 0.028 | -0.003 to 0.059 | 0.080 | | N/A | N/A |
| Passive-reacting coping | 0.211 | 0.165 to 0.258 | **<0.001** | 0.209 | 0.174 to 0.243 | **<0.001** | | 0.007 | 0.931 |
| Palliative-reacting coping | -0.016 | -0.055 to 0.024 | 0.434 | 0.008 | -0.021 to 0.037 | 0.599 | | N/A | N/A |
| Avoidance coping | -0.009 | -0.048 to 0.029 | 0.629 | -0.010 | -0.046 to 0.025 | 0.574 | | N/A | N/A |

Supplementary Table 6 - Secondary analyses of coping strategies on CAPE subdomains in mutually adjusted models

* p value threshold was accepted as <0.016 for 3 mutually adjusted models including all coping styles on 3 CAPE subdomains

|  | **Males** | | | | **Females** | | | | **Sex Differences** | |
| --- | --- | --- | --- | --- | --- | --- | --- | --- | --- | --- |
|  | Beta | 95% CI | *p*-value **^a^** | Adjusted R^2^**^b^** | Beta | 95% CI | *p*-value **^a^** | Adjusted R^2^**^b^** | χ² | *p*-value |
| **CAPE positive** | | | | | | | | | | |
| Active coping | 0.001 | -0.030 to 0.031 | 0.960 | 0.1% | -0.002 | -0.033 to 0.029 | 0.889 | 5.6% | N/A | N/A |
| Seeking social support | 0.040 | 0.004 to 0.076 | 0.028 | 1.5% | -0.042 | -0.077 to -0.008 | 0.016 | 7.9% | N/A | N/A |
| Reassuring thoughts | 0.001 | -0.039 to 0.040 | 0.980 | 0.1% | -0.007 | -0.041 to 0.027 | 0.686 | 5.7% | N/A | N/A |
| Emotional expression | 0.015 | -0.017 to 0.047 | 0.369 | 0.4% | 0.046 | 0.019 to 0.073 | **<0.001** | 8.3% | 2.1 | 0.147 |
| Passive-reacting coping | 0.101 | 0.055 to 0.147 | **<0.001** | 11.3% | 0.102 | 0.068 to 0.135 | **<0.001** | 19.5% | 0.001 | 0.981 |
| Palliative-reacting coping | 0.018 | -0.020 to 0.055 | 0.350 | 0.4% | 0.029 | 0.002 to 0.057 | 0.036 | 6.8% | N/A | N/A |
| Avoidance coping | 0.020 | -0.013 to 0.053 | 0.229 | 0.6% | 0.041 | 0.007 to 0.075 | 0.019 | 7.7% | N/A | N/A |
| **CAPE negative** | | | | | | | | | | |
| Active coping | -0.050 | -0.091 to -0.009 | 0.017 | 2.1% | -0.045 | -0.080 to -0.010 | 0.012 | 1.6% | N/A | N/A |
| Seeking social support | -0.022 | -0.072 to 0.028 | 0.385 | 0.2% | -0.078 | -0.112 to -0.044 | **<0.001** | 5.3% | 3.34 | 0.068 |
| Reassuring thoughts | 0.007 | -0.034 to 0.047 | 0.744 | -0.002%* | -0.034 | -0.069 to 0.001 | 0.055 | 1.0% | N/A | N/A |
| Emotional expression | -0.024 | -0.064 to 0.016 | 0.239 | 0.4% | 0.044 | 0.006 to 0.081 | 0.022 | 1.7% | N/A | N/A |
| Passive-reacting coping | 0.169 | 0.125 to 0.213 | **<0.001** | 19.6% | 0.166 | 0.133 to 0.199 | **<0.001** | 25.8% | 0.01 | 0.911 |
| Palliative-reacting coping | 0.025 | -0.021 to 0.072 | 0.282 | 0.4% | 0.014 | -0.014 to 0.042 | 0.337 | 0.2% | N/A | N/A |
| Avoidance coping | 0.119 | 0.073 to 0.165 | **<0.001** | 11.5% | 0.095 | 0.057 to 0.133 | **<0.001** | 7.8% | 0.61 | 0.435 |
| **CAPE depressive** | | | | | | | | | | |
| Active coping | -0.031 | -0.071 to 0.009 | 0.122 | 0.5% | -0.074 | -0.113 to -0.035 | **<0.001** | 3.5% | 2.36 | 0.124 |
| Seeking social support | 0.047 | -0.003 to 0.097 | 0.065 | 0.8% | -0.033 | -0.076 to 0.010 | 0.131 | 0.8% | N/A | N/A |
| Reassuring thoughts | -0.025 | -0.067 to 0.017 | 0.246 | 0.1% | -0.038 | -0.076 to 0.000 | 0.052 | 1.0% | N/A | N/A |
| Emotional expression | 0.028 | -0.013 to 0.069 | 0.182 | 0.2% | 0.081 | 0.040 to 0.121 | **<0.001** | 4.7% | 3.35 | 0.067 |
| Passive-reacting coping | 0.206 | 0.163 to 0.250 | **<0.001** | 32.1% | 0.219 | 0.181 to 0.257 | **<0.001** | 36.9% | 0.2 | 0.658 |
| Palliative-reacting coping | 0.028 | -0.017 to 0.074 | 0.221 | 0.1% | 0.037 | 0.005 to 0.068 | 0.021 | 1.1% | N/A | N/A |
| Avoidance coping | 0.045 | -0.002 to 0.093 | 0.063 | 1.4% | 0.067 | 0.024 to 0.110 | 0.003 | 3.2% | N/A | N/A |

Supplementary Table 7 - Sensitivity analyses of coping styles on CAPE subdomains in independent models

**^a^** p value threshold was accepted as <0.0023 for 21 independent models for 7 coping styles on 3 CAPE subdomains.

**^b^** Adjusted R^2^ values are presented to demonstrate the proportion of variance explained by each respective model including adjustments for age and family relatedness.

*Adjusted R2 value was negative, indicating that the included variables explained less variance in psychosis expression than the mean-only model.

| Supplementary Table-8: Interaction between childhood adversity subtypes and coping strategies on CAPE total | | | | | | | | |
| --- | --- | --- | --- | --- | --- | --- | --- | --- |
|  | **Males** | | | **Females** | | | **Sex Differences** | |
| **Interaction** | Beta | 95% CI | *p** | Beta | 95% CI | *p** | χ² | *p* |
| **Active coping** | | | | | | | | |
| Total CA | -0.004 | -0.05 to 0.04 | 0.866 | -0.01 | -0.04 to 0.02 | 0.587 | N/A | N/A |
| Emotional abuse | -0.02 | -0.09 to 0.04 | 0.473 | 0.004 | -0.06 to 0.07 | 0.893 | N/A | N/A |
| Physical abuse | 0.05 | -0.21 to 0.32 | 0.698 | -0.19 | -0.40 to 0.03 | 0.085 | N/A | N/A |
| Sexual abuse | -0.11 | -0.39 to 0.16 | 0.414 | -0.01 | -0.11 to 0.08 | 0.762 | N/A | N/A |
| Emotional neglect | -0.03 | -0.08 to 0.03 | 0.337 | -0.02 | -0.07 to 0.03 | 0.457 | N/A | N/A |
| Physical neglect | -0.01 | -0.10 to 0.09 | 0.909 | -0.04 | -0.13 to 0.05 | 0.360 | N/A | N/A |
| **Seeking social support** |  |  |  |  |  |  |  |  |
| Total CA | 0.01 | -0.04 to 0.06 | 0.673 | -0.04 | -0.06 to -0.02 | **<0.001** | 3.43 | 0.064 |
| Emotional abuse | -0.02 | -0.11 to 0.06 | 0.599 | -0.10 | -0.17 to -0.03 | **0.003** | 2.18 | 0.14 |
| Physical abuse | 0.17 | -0.02 to 0.35 | 0.078 | -0.32 | -0.54 to -0.10 | **0.005** | 13.19 | **<0.001** |
| Sexual abuse | -0.04 | -0.24 to 0.16 | 0.708 | -0.07 | -0.23 to 0.09 | 0.370 | N/A | N/A |
| Emotional neglect | 0.00 | -0.07 to 0.07 | 0.917 | -0.06 | -0.13 to 0.01 | 0.071 | 1.94 | 0.16 |
| Physical neglect | 0.08 | -0.03 to 0.20 | 0.154 | -0.14 | -0.24 to -0.05 | **0.002** | 9.01 | **0.003** |
| **Reassuring thoughts** |  |  |  |  |  |  |  |  |
| Total CA | -0.01 | -0.05 to 0.03 | 0.535 | -0.03 | -0.04 to -0.01 | **0.001** | 0.35 | 0.56 |
| Emotional abuse | -0.05 | -0.11 to 0.01 | 0.126 | -0.05 | -0.12 to 0.02 | 0.162 | N/A | N/A |
| Physical abuse | 0.07 | -0.09 to 0.22 | 0.408 | -0.22 | -0.37 to -0.07 | **0.005** | 7.32 | **0.01** |
| Sexual abuse | 0.09 | -0.03 to 0.21 | 0.127 | -0.08 | -0.22 to 0.05 | 0.223 | N/A | N/A |
| Emotional neglect | -0.09 | -0.15 to -0.04 | **0.001** | -0.07 | -0.14 to -0.01 | **0.027** | 0.20 | 0.65 |
| Physical neglect | 0.01 | -0.08 to 0.11 | 0.772 | -0.17 | -0.27 to -0.07 | **0.001** | 6.88 | **0.01** |
| **Emotional expression** |  |  |  |  |  |  |  |  |
| Total CA | -0.02 | -0.05 to 0.01 | 0.109 | 0.01 | -0.04 to 0.05 | 0.764 | N/A | N/A |
| Emotional abuse | -0.06 | -0.13 to 0.00 | 0.068 | 0.01 | -0.06 to 0.08 | 0.721 | N/A | N/A |
| Physical abuse | -0.11 | -0.24 to 0.02 | 0.087 | -0.01 | -0.23 to 0.21 | 0.954 | N/A | N/A |
| Sexual abuse | -0.08 | -0.24 to 0.08 | 0.340 | 0.14 | -0.01 to 0.28 | 0.064 | N/A | N/A |
| Emotional neglect | -0.01 | -0.07 to 0.05 | 0.664 | 0.01 | -0.05 to 0.07 | 0.659 | N/A | N/A |
| Physical neglect | 0.01 | -0.09 to 0.11 | 0.860 | 0.09 | -0.03 to 0.20 | 0.141 | N/A | N/A |
| **Passive-reacting coping** |  |  |  |  |  |  |  |  |
| Total CA | 0.02 | -0.01 to 0.04 | 0.231 | 0.04 | 0.01 to 0.06 | **0.002** | 1.27 | 0.26 |
| Emotional abuse | 0.00 | -0.07 to 0.06 | 0.941 | 0.06 | -0.01 to 0.12 | 0.071 | N/A | N/A |
| Physical abuse | 0.08 | -0.06 to 0.22 | 0.266 | 0.14 | -0.13 to 0.41 | 0.298 | N/A | N/A |
| Sexual abuse | 0.05 | -0.11 to 0.21 | 0.532 | 0.04 | -0.11 to 0.19 | 0.566 | N/A | N/A |
| Emotional neglect | 0.04 | -0.03 to 0.11 | 0.213 | 0.07 | 0.02 to 0.12 | **0.008** | 0.36 | 0.55 |
| Physical neglect | 0.08 | 0.01 to 0.15 | **0.031** | 0.07 | -0.03 to 0.16 | 0.157 | 0.03 | 0.86 |
| **Palliative-reacting coping** |  |  |  |  |  |  |  |  |
| Total CA | -0.003 | -0.05 to 0.04 | 0.873 | -0.03 | -0.05 to -0.02 | **<0.001** | 1.36 | 0.243 |
| Emotional abuse | -0.06 | -0.15 to 0.03 | 0.166 | -0.04 | -0.09 to 0.01 | 0.150 | N/A | N/A |
| Physical abuse | 0.05 | -0.21 to 0.31 | 0.683 | -0.21 | -0.37 to -0.05 | **0.010** | 3.16 | 0.08 |
| Sexual abuse | -0.11 | -0.28 to 0.06 | 0.200 | -0.08 | -0.21 to 0.06 | 0.248 | N/A | N/A |
| Emotional neglect | -0.04 | -0.11 to 0.03 | 0.289 | -0.03 | -0.08 to 0.02 | 0.187 | N/A | N/A |
| Physical neglect | 0.09 | -0.05 to 0.22 | 0.225 | -0.10 | -0.17 to -0.02 | **0.018** | 5.42 | **0.02** |
| **Avoidance coping** |  |  |  |  |  |  |  |  |
| Total CA | 0.02 | 0.00 to 0.04 | **0.048** | -0.004 | -0.04 to 0.03 | 0.783 | 1.55 | 0.21 |
| Emotional abuse | -0.02 | -0.10 to 0.06 | 0.556 | 0.04 | -0.04 to 0.12 | 0.336 | N/A | N/A |
| Physical abuse | 0.20 | 0.13 to 0.27 | **<0.001** | -0.08 | -0.32 to 0.15 | 0.498 | 4.90 | **0.03** |
| Sexual abuse | 0.27 | 0.10 to 0.44 | **0.002** | -0.10 | -0.23 to 0.03 | 0.146 | 11.47 | **<0.001** |
| Emotional neglect | 0.04 | -0.02 to 0.11 | 0.202 | -0.02 | -0.09 to 0.04 | 0.486 | N/A | N/A |
| Physical neglect | 0.06 | -0.05 to 0.18 | 0.282 | -0.02 | -0.13 to 0.08 | 0.674 | N/A | N/A |

** Bonferroni correction was not applied for the interaction analyses, as these findings are exploratory in nature*

Supplementary Table-9: Interaction between childhood adversity subtypes and coping strategies on CAPE positive

|  | **Males** | | | **Females** | | | **Sex Differences** | |
| --- | --- | --- | --- | --- | --- | --- | --- | --- |
| **Interaction** | Beta | 95% CI | *p** | Beta | 95% CI | *p** | χ² | *p*-value |
| **Active coping** | | | | | | | | |
| Total CA | -0.03 | -0.06 to 0.01 | 0.110 | -0.02 | -0.05 to 0.02 | 0.286 | N/A | N/A |
| Emotional abuse | -0.04 | -0.10 to 0.03 | 0.308 | 0.01 | -0.06 to 0.09 | 0.698 | N/A | N/A |
| Physical abuse | -0.03 | -0.25 to 0.18 | 0.760 | -0.25 | -0.42 to -0.08 | **0.004** | 2.55 | 0.11 |
| Sexual abuse | -0.17 | -0.45 to 0.12 | 0.244 | 0.04 | -0.08 to 0.16 | 0.499 | N/A | N/A |
| Emotional neglect | -0.05 | -0.11 to 0.01 | 0.088 | -0.03 | -0.09 to 0.03 | 0.386 | N/A | N/A |
| Physical neglect | -0.04 | -0.13 to 0.06 | 0.454 | -0.06 | -0.15 to 0.02 | 0.144 | N/A | N/A |
| **Seeking social support** |  |  |  |  |  |  |  |  |
| Total CA | 0.01 | -0.03 to 0.06 | 0.521 | -0.05 | -0.07 to -0.03 | **<0.001** | 8.10 | **0.004** |
| Emotional abuse | 0.00 | -0.09 to 0.09 | 0.979 | -0.10 | -0.18 to -0.03 | **0.008** | 3.18 | 0.07 |
| Physical abuse | 0.14 | -0.04 to 0.32 | 0.136 | -0.38 | -0.60 to -0.16 | **0.001** | 13.70 | **<0.001** |
| Sexual abuse | -0.06 | -0.26 to 0.15 | 0.595 | -0.07 | -0.24 to 0.10 | 0.397 | N/A | N/A |
| Emotional neglect | 0.01 | -0.07 to 0.08 | 0.891 | -0.07 | -0.15 to 0.00 | 0.055 | N/A | N/A |
| Physical neglect | 0.08 | -0.06 to 0.22 | 0.242 | -0.16 | -0.26 to -0.06 | **0.002** | 7.51 | **0.01** |
| **Reassuring thoughts** |  |  |  |  |  |  |  |  |
| Total CA | -0.01 | -0.05 to 0.03 | 0.655 | -0.03 | -0.05 to -0.02 | **<0.001** | 1.03 | 0.31 |
| Emotional abuse | -0.05 | -0.13 to 0.02 | 0.140 | -0.05 | -0.12 to 0.03 | 0.238 | N/A | N/A |
| Physical abuse | 0.09 | -0.09 to 0.28 | 0.321 | -0.31 | -0.46 to -0.15 | **<0.001** | 11.71 | **<0.001** |
| Sexual abuse | 0.08 | -0.05 to 0.21 | 0.245 | -0.06 | -0.23 to 0.11 | 0.485 | N/A | N/A |
| Emotional neglect | -0.07 | -0.14 to 0.00 | 0.057 | -0.04 | -0.11 to 0.03 | 0.298 | N/A | N/A |
| Physical neglect | 0.02 | -0.08 to 0.13 | 0.653 | -0.17 | -0.29 to -0.05 | **0.007** | 5.65 | **0.02** |
| **Emotional expression** |  |  |  |  |  |  |  |  |
| Total CA | -0.02 | -0.04 to 0.01 | 0.206 | -0.01 | -0.05 to 0.03 | 0.619 | N/A | N/A |
| Emotional abuse | -0.06 | -0.14 to 0.01 | 0.074 | -0.02 | -0.09 to 0.05 | 0.646 | N/A | N/A |
| Physical abuse | -0.10 | -0.20 to 0.00 | **0.049** | -0.02 | -0.23 to 0.19 | 0.830 | 0.43 | 0.51 |
| Sexual abuse | -0.12 | -0.27 to 0.03 | 0.110 | 0.15 | 0.02 to 0.28 | **0.027** | 7.25 | **0.01** |
| Emotional neglect | -0.02 | -0.09 to 0.04 | 0.439 | -0.01 | -0.07 to 0.05 | 0.745 | N/A | N/A |
| Physical neglect | 0.03 | -0.07 to 0.13 | 0.582 | 0.03 | -0.07 to 0.14 | 0.560 | N/A | N/A |
| **Passive-reacting coping** |  |  |  |  |  |  |  |  |
| Total CA | 0.02 | -0.01 to 0.05 | 0.170 | 0.04 | 0.01 to 0.07 | **0.004** | 1.20 | 0.27 |
| Emotional abuse | -0.02 | -0.11 to 0.07 | 0.632 | 0.03 | -0.05 to 0.10 | 0.506 | N/A | N/A |
| Physical abuse | 0.08 | -0.05 to 0.22 | 0.218 | 0.21 | -0.04 to 0.46 | 0.101 | N/A | N/A |
| Sexual abuse | 0.07 | -0.13 to 0.26 | 0.487 | 0.05 | -0.11 to 0.20 | 0.570 | N/A | N/A |
| Emotional neglect | 0.04 | -0.04 to 0.13 | 0.318 | 0.08 | 0.01 to 0.14 | **0.018** | 0.41 | 0.52 |
| Physical neglect | 0.12 | 0.03 to 0.21 | **0.011** | 0.08 | -0.01 to 0.17 | 0.079 | 0.32 | 0.57 |
| **Palliative-reacting coping** |  |  |  |  |  |  |  |  |
| Total CA | -0.03 | -0.06 to 0.01 | 0.205 | -0.04 | -0.06 to -0.02 | **<0.001** | 0.58 | 0.45 |
| Emotional abuse | -0.08 | -0.18 to 0.01 | 0.081 | -0.02 | -0.09 to 0.04 | 0.473 | N/A | N/A |
| Physical abuse | -0.03 | -0.25 to 0.19 | 0.783 | -0.26 | -0.44 to -0.08 | **0.004** | 2.75 | 0.10 |
| Sexual abuse | -0.11 | -0.31 to 0.09 | 0.265 | -0.03 | -0.19 to 0.13 | 0.725 | N/A | N/A |
| Emotional neglect | -0.06 | -0.14 to 0.01 | 0.078 | -0.04 | -0.09 to 0.02 | 0.212 | N/A | N/A |
| Physical neglect | 0.08 | -0.03 to 0.20 | 0.158 | -0.11 | -0.19 to -0.02 | **0.016** | 7.00 | **0.01** |
| **Avoidance coping** |  |  |  |  |  |  |  |  |
| Total CA | 0.01 | -0.01 to 0.03 | 0.501 | -0.003 | -0.04 to 0.03 | 0.833 | N/A | N/A |
| Emotional abuse | -0.07 | -0.15 to 0.01 | 0.099 | 0.04 | -0.05 to 0.12 | 0.380 | N/A | N/A |
| Physical abuse | 0.17 | 0.05 to 0.29 | **0.005** | -0.06 | -0.30 to 0.19 | 0.653 | 2.67 | 0.10 |
| Sexual abuse | 0.22 | -0.01 to 0.46 | 0.062 | -0.02 | -0.18 to 0.14 | 0.822 | N/A | N/A |
| Emotional neglect | 0.04 | -0.03 to 0.10 | 0.242 | -0.01 | -0.08 to 0.06 | 0.711 | N/A | N/A |
| Physical neglect | 0.06 | -0.06 to 0.18 | 0.322 | 0.01 | -0.09 to 0.11 | 0.844 | N/A | N/A |

** Bonferroni correction was not applied for the interaction analyses, as these findings are exploratory in nature*

Supplementary Table-10: Interaction between childhood adversity subtypes and coping strategies on CAPE negative

|  | **Males** | | | **Females** | | | **Sex Differences** | |
| --- | --- | --- | --- | --- | --- | --- | --- | --- |
| **Interaction** | Beta | 95% CI | *p** | Beta | 95% CI | *p** | χ² | *p* |
| **Active coping** | | | | | | | | |
| Total CA | 0.02 | -0.05 to 0.08 | 0.616 | -0.003 | -0.04 to 0.04 | 0.877 | N/A | N/A |
| Emotional abuse | -0.03 | -0.12 to 0.06 | 0.563 | 0.01 | -0.07 to 0.09 | 0.866 | N/A | N/A |
| Physical abuse | 0.17 | -0.15 to 0.48 | 0.300 | -0.19 | -0.43 to 0.05 | 0.122 | N/A | N/A |
| Sexual abuse | -0.06 | -0.36 to 0.23 | 0.665 | -0.08 | -0.18 to 0.02 | 0.107 | N/A | N/A |
| Emotional neglect | 0.00 | -0.07 to 0.08 | 0.904 | 0.00 | -0.07 to 0.07 | 0.896 | N/A | N/A |
| Physical neglect | 0.02 | -0.09 to 0.14 | 0.707 | -0.05 | -0.16 to 0.06 | 0.408 | N/A | N/A |
| **Seeking social support** |  |  |  |  |  |  |  |  |
| Total CA | 0.001 | -0.07 to 0.08 | 0.958 | -0.03 | -0.05 to -0.01 | **0.006** | 0.73 | 0.39 |
| Emotional abuse | -0.05 | -0.17 to 0.07 | 0.405 | -0.10 | -0.17 to -0.02 | **0.008** | 0.38 | 0.54 |
| Physical abuse | 0.22 | -0.02 to 0.46 | 0.069 | -0.26 | -0.52 to 0.01 | 0.058 | N/A | N/A |
| Sexual abuse | 0.02 | -0.22 to 0.26 | 0.883 | -0.10 | -0.27 to 0.07 | 0.239 | N/A | N/A |
| Emotional neglect | 0.00 | -0.10 to 0.10 | 0.942 | -0.04 | -0.11 to 0.03 | 0.213 | N/A | N/A |
| Physical neglect | 0.07 | -0.05 to 0.20 | 0.247 | -0.14 | -0.24 to -0.04 | **0.008** | 6.51 | **0.01** |
| **Reassuring thoughts** |  |  |  |  |  |  |  |  |
| Total CA | -0.02 | -0.08 to 0.03 | 0.397 | -0.03 | -0.05 to -0.01 | **0.01** | 0.01 | 0.91 |
| Emotional abuse | -0.06 | -0.14 to 0.02 | 0.117 | -0.03 | -0.11 to 0.05 | 0.453 | N/A | N/A |
| Physical abuse | 0.06 | -0.10 to 0.22 | 0.485 | -0.15 | -0.34 to 0.04 | 0.124 | N/A | N/A |
| Sexual abuse | 0.09 | -0.05 to 0.24 | 0.219 | -0.12 | -0.26 to 0.02 | 0.087 | N/A | N/A |
| Emotional neglect | -0.13 | -0.20 to -0.06 | **<0.001** | -0.11 | -0.18 to -0.04 | **0.002** | 0.12 | 0.73 |
| Physical neglect | -0.01 | -0.10 to 0.09 | 0.899 | -0.19 | -0.29 to -0.08 | **0.001** | 6.23 | **0.01** |
| **Emotional expression** |  |  |  |  |  |  |  |  |
| Total CA | -0.03 | -0.07 to 0.01 | 0.134 | 0.02 | -0.03 to 0.07 | 0.346 | N/A | N/A |
| Emotional abuse | -0.07 | -0.16 to 0.02 | 0.107 | 0.04 | -0.05 to 0.12 | 0.423 | N/A | N/A |
| Physical abuse | -0.13 | -0.30 to 0.04 | 0.125 | 0.00 | -0.24 to 0.23 | 0.970 | N/A | N/A |
| Sexual abuse | -0.06 | -0.27 to 0.15 | 0.592 | 0.10 | -0.10 to 0.29 | 0.329 | N/A | N/A |
| Emotional neglect | 0.00 | -0.08 to 0.07 | 0.942 | 0.03 | -0.05 to 0.11 | 0.434 | N/A | N/A |
| Physical neglect | 0.00 | -0.12 to 0.12 | 0.978 | 0.14 | -0.01 to 0.28 | 0.069 | N/A | N/A |
| **Passive-reacting coping** |  |  |  |  |  |  |  |  |
| Total CA | 0.01 | -0.04 to 0.06 | 0.710 | 0.03 | 0.01 to 0.06 | **0.019** | 0.84 | 0.36 |
| Emotional abuse | 0.02 | -0.07 to 0.11 | 0.678 | 0.09 | 0.02 to 0.16 | **0.017** | 1.22 | 0.27 |
| Physical abuse | 0.06 | -0.16 to 0.28 | 0.616 | 0.11 | -0.19 to 0.41 | 0.480 | N/A | N/A |
| Sexual abuse | 0.06 | -0.09 to 0.21 | 0.423 | 0.02 | -0.16 to 0.20 | 0.837 | N/A | N/A |
| Emotional neglect | 0.02 | -0.08 to 0.12 | 0.651 | 0.06 | 0.00 to 0.12 | 0.056 | N/A | N/A |
| Physical neglect | 0.02 | -0.08 to 0.13 | 0.640 | 0.06 | -0.06 to 0.18 | 0.328 | N/A | N/A |
| **Palliative-reacting coping** |  |  |  |  |  |  |  |  |
| Total CA | 0.01 | -0.05 to 0.07 | 0.710 | -0.03 | -0.05 to -0.01 | **0.006** | 1.35 | 0.24 |
| Emotional abuse | -0.06 | -0.17 to 0.05 | 0.280 | -0.05 | -0.11 to 0.01 | 0.134 | N/A | N/A |
| Physical abuse | 0.15 | -0.14 to 0.44 | 0.306 | -0.14 | -0.32 to 0.03 | 0.111 | N/A | N/A |
| Sexual abuse | -0.08 | -0.23 to 0.07 | 0.309 | -0.13 | -0.27 to 0.00 | 0.051 | N/A | N/A |
| Emotional neglect | -0.02 | -0.11 to 0.07 | 0.709 | -0.03 | -0.08 to 0.03 | 0.359 | N/A | N/A |
| Physical neglect | 0.08 | -0.12 to 0.29 | 0.431 | -0.10 | -0.19 to -0.01 | **0.035** | 2.52 | 0.11 |
| **Avoidance coping** |  |  |  |  |  |  |  |  |
| Total CA | 0.02 | 0.00 to 0.05 | **0.049** | -0.01 | -0.05 to 0.03 | 0.580 | 2.38 | 0.12 |
| Emotional abuse | 0.03 | -0.07 to 0.13 | 0.606 | 0.05 | -0.04 to 0.13 | 0.313 | N/A | N/A |
| Physical abuse | 0.21 | 0.14 to 0.27 | **<0.001** | -0.08 | -0.35 to 0.19 | 0.560 | 4.21 | **0.04** |
| Sexual abuse | 0.29 | 0.15 to 0.43 | **<0.001** | -0.15 | -0.29 to -0.01 | **0.034** | 19.43 | **<0.001** |
| Emotional neglect | 0.04 | -0.05 to 0.13 | 0.347 | -0.03 | -0.11 to 0.05 | 0.412 | N/A | N/A |
| Physical neglect | 0.05 | -0.07 to 0.18 | 0.406 | -0.05 | -0.19 to 0.09 | 0.450 | N/A | N/A |

** Bonferroni correction was not applied for the interaction analyses, as these findings are exploratory in nature*

Supplementary Table-11: Interaction between childhood adversity subtypes and coping strategies on CAPE depressive

|  | **Males** | | | **Females** | | | **Sex Differences** | |
| --- | --- | --- | --- | --- | --- | --- | --- | --- |
| **Interaction** | Beta | 95% CI | *p** | Beta | 95% CI | *p** | χ² | *p* |
| **Active coping** | | | | | | | | |
| Total CA | 0.02 | -0.04 to 0.09 | 0.525 | 0.003 | -0.05 to 0.06 | 0.901 | N/A | N/A |
| Emotional abuse | 0.01 | -0.07 to 0.10 | 0.778 | -0.02 | -0.12 to 0.07 | 0.609 | N/A | N/A |
| Physical abuse | 0.07 | -0.30 to 0.43 | 0.717 | -0.04 | -0.38 to 0.31 | 0.839 | N/A | N/A |
| Sexual abuse | -0.07 | -0.35 to 0.21 | 0.643 | -0.03 | -0.17 to 0.10 | 0.624 | N/A | N/A |
| Emotional neglect | -0.02 | -0.09 to 0.05 | 0.561 | -0.03 | -0.11 to 0.04 | 0.421 | N/A | N/A |
| Physical neglect | 0.02 | -0.11 to 0.15 | 0.744 | 0.02 | -0.10 to 0.15 | 0.694 | N/A | N/A |
| **Seeking social support** |  |  |  |  |  |  |  |  |
| Total CA | 0.02 | -0.06 to 0.09 | 0.639 | -0.02 | -0.06 to 0.02 | 0.228 | N/A | N/A |
| Emotional abuse | -0.03 | -0.15 to 0.09 | 0.606 | -0.12 | -0.21 to -0.02 | **0.015** | 1.28 | 0.26 |
| Physical abuse | 0.14 | -0.12 to 0.41 | 0.281 | -0.29 | -0.52 to -0.07 | **0.010** | 8.82 | **0.003** |
| Sexual abuse | -0.09 | -0.29 to 0.11 | 0.371 | -0.02 | -0.19 to 0.16 | 0.868 | N/A | N/A |
| Emotional neglect | 0.01 | -0.08 to 0.11 | 0.789 | -0.07 | -0.17 to 0.02 | 0.117 | N/A | N/A |
| Physical neglect | 0.11 | -0.06 to 0.27 | 0.215 | -0.12 | -0.23 to -0.01 | **0.034** | 4.91 | **0.03** |
| **Reassuring thoughts** |  |  |  |  |  |  |  |  |
| Total CA | -0.004 | -0.06 to 0.06 | 0.894 | -0.01 | -0.04 to 0.02 | 0.529 | N/A | N/A |
| Emotional abuse | -0.01 | -0.09 to 0.07 | 0.812 | -0.08 | -0.16 to 0.00 | **0.042** | 1.67 | 0.20 |
| Physical abuse | 0.01 | -0.18 to 0.21 | 0.906 | -0.13 | -0.30 to 0.04 | 0.146 | N/A | N/A |
| Sexual abuse | 0.13 | -0.03 to 0.30 | 0.116 | -0.08 | -0.22 to 0.05 | 0.236 | N/A | N/A |
| Emotional neglect | -0.09 | -0.17 to -0.02 | **0.019** | -0.09 | -0.17 to -0.01 | **0.032** | 0.00 | 0.97 |
| Physical neglect | 0.02 | -0.12 to 0.16 | 0.769 | -0.13 | -0.24 to -0.03 | **0.013** | 3.20 | 0.07 |
| **Emotional expression** |  |  |  |  |  |  |  |  |
| Total CA | -0.03 | -0.09 to 0.03 | 0.300 | 0.02 | -0.04 to 0.08 | 0.463 | N/A | N/A |
| Emotional abuse | -0.04 | -0.13 to 0.06 | 0.467 | 0.05 | -0.05 to 0.14 | 0.361 | N/A | N/A |
| Physical abuse | -0.12 | -0.34 to 0.11 | 0.309 | 0.03 | -0.23 to 0.29 | 0.816 | N/A | N/A |
| Sexual abuse | -0.01 | -0.21 to 0.19 | 0.891 | 0.17 | -0.02 to 0.35 | 0.076 | N/A | N/A |
| Emotional neglect | 0.00 | -0.09 to 0.08 | 0.935 | 0.04 | -0.05 to 0.13 | 0.380 | N/A | N/A |
| Physical neglect | -0.02 | -0.17 to 0.13 | 0.784 | 0.14 | -0.02 to 0.30 | 0.076 | N/A | N/A |
| **Passive-reacting coping** |  |  |  |  |  |  |  |  |
| Total CA | 0.02 | -0.02 to 0.07 | 0.317 | 0.03 | -0.003 to 0.06 | 0.076 | N/A | N/A |
| Emotional abuse | 0.00 | -0.08 to 0.09 | 0.915 | 0.08 | 0.01 to 0.16 | **0.032** | 1.74 | 0.19 |
| Physical abuse | 0.10 | -0.08 to 0.28 | 0.288 | 0.04 | -0.28 to 0.36 | 0.824 | N/A | N/A |
| Sexual abuse | -0.02 | -0.14 to 0.10 | 0.753 | 0.08 | -0.08 to 0.25 | 0.327 | N/A | N/A |
| Emotional neglect | 0.08 | 0.00 to 0.17 | **0.046** | 0.07 | 0.00 to 0.14 | 0.065 | 0.10 | 0.75 |
| Physical neglect | 0.06 | -0.05 to 0.18 | 0.294 | 0.04 | -0.09 to 0.17 | 0.561 | N/A | N/A |
| **Palliative-reacting coping** |  |  |  |  |  |  |  |  |
| Total CA | 0.02 | -0.05 to 0.10 | 0.551 | -0.03 | -0.06 to 0.004 | 0.095 | N/A | N/A |
| Emotional abuse | -0.01 | -0.13 to 0.10 | 0.834 | -0.07 | -0.13 to 0.00 | **0.038** | 0.78 | 0.38 |
| Physical abuse | 0.10 | -0.28 to 0.48 | 0.619 | -0.20 | -0.35 to -0.05 | **0.007** | 2.34 | 0.13 |
| Sexual abuse | -0.15 | -0.35 to 0.05 | 0.152 | -0.11 | -0.23 to 0.02 | 0.101 | N/A | N/A |
| Emotional neglect | 0.00 | -0.10 to 0.09 | 0.917 | -0.04 | -0.11 to 0.03 | 0.227 | N/A | N/A |
| Physical neglect | 0.10 | -0.11 to 0.30 | 0.352 | -0.06 | -0.15 to 0.03 | 0.169 | N/A | N/A |
| **Avoidance coping** |  |  |  |  |  |  |  |  |
| Total CA | 0.04 | 0.01 to 0.07 | **0.010** | 0.004 | -0.03 to 0.04 | 0.827 | 2.38 | 0.12 |
| Emotional abuse | 0.00 | -0.10 to 0.11 | 0.928 | 0.03 | -0.07 to 0.13 | 0.530 | N/A | N/A |
| Physical abuse | 0.26 | 0.14 to 0.37 | **<0.001** | -0.15 | -0.37 to 0.07 | 0.180 | 9.82 | **<0.001** |
| Sexual abuse | 0.34 | 0.17 to 0.52 | **<0.001** | -0.19 | -0.32 to -0.06 | **0.003** | 24.14 | **<0.001** |
| Emotional neglect | 0.05 | -0.05 to 0.14 | 0.333 | -0.03 | -0.12 to 0.06 | 0.519 | N/A | N/A |
| Physical neglect | 0.10 | -0.10 to 0.30 | 0.328 | -0.05 | -0.20 to 0.09 | 0.468 | N/A | N/A |

** Bonferroni correction was not applied for the interaction analyses, as these findings are exploratory in nature*
